# Two Cases of Atraumatic Chylous Ascites Characterized by Hypotriglyceridemia and Partially Managed with an Oral Fat-Free Elemental Diet

**DOI:** 10.1155/2020/7641476

**Published:** 2020-01-07

**Authors:** Michihiro Kamigaki, Satoko Kunita, Makoto Nakano, Miwako Tanaka, Shinya Aoki, Katsutoshi Tsuga, Hiroyuki Ito, Hideo Matsuura

**Affiliations:** Department of Internal Medicine, Saiseikai Kure Hospital, 2-1-13 Sanjo, Kure City, Hiroshima Prefecture 737-0821, Japan

## Abstract

Most cases of chylous ascites occur after surgery, but it also develops in nonoperative cases, although rarely. Such cases are often difficult to treat. In this study, we treated 2 cases of atraumatic chylous ascites, which were controlled by combining diuretic treatment with an oral fat-free elemental diet (Elental®, EA Pharma Co., Ltd., Tokyo, Japan). Elental can provide oral nutrition compatible with a lipid-restricted diet, which may be useful for control of chylous ascites. We report on these cases, including literature review-based considerations.

## 1. Introduction

Chylous ascites is characterized by the accumulation of triglyceride (TG)-rich peritoneal fluid with a milky appearance due to intestinal tract and the mesentery leakage [1]. Most cases of chylous ascites occur after surgery [1–3], and the proportion of nonsurgical cases is low. The most frequent causes of nonsurgical chylous ascites include malignancies (25%), liver cirrhosis (16%), and tuberculosis (15%) [4, 5]. Chylous ascites may be treated by ligation of broken lymphatic vessels after surgery. It is generally recommended to place a central venous catheter in a fasting patient as a means of delivering total parenteral nutrition (TPN) and bypassing the intestinal tract [2, 4, 6]. However, parenteral nutrition together with an oral fat-free elemental diet have reportedly been used as initial treatments for chylous ascites [7, 8]. As there are cases where TPN cannot be used for some reason, elemental diet can also be an option for such cases. Here we present two cases of atraumatic chylous ascites, which were controlled by combining diuretic treatment with an elemental diet. In both cases, the patients provided informed consent.

## 2. Case Presentations

### 2.1. Case 1

**Figure 1:**
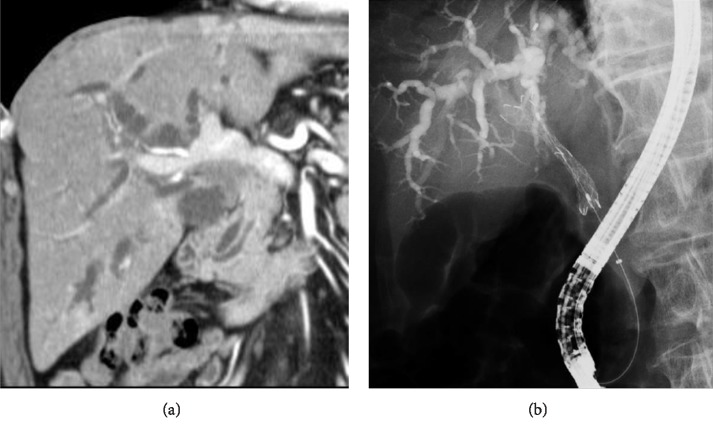
The stenosis and contrast effect are recognized in the bile duct of the hepatic portal. Expansion of bilateral intrahepatic bile duct is admitted upstream of stenosis (a). ERCP was performed and a metallic stent was placed in the left and right hepatic bile ducts (b).

An 83-year-old man with a medical history of high blood pressure and a gastric ulcer complained of epigastralgia and symptoms of jaundice, and was admitted to our hospital for observation. He was diagnosed with hepatic portal cholangiocarcinoma. Given the patient's age and the progression of the lesion, radical curative resection was considered too difficult. Eventually, he was discharged after undergoing an endoscopic biliary stent placement procedure (Figure 1).

At an outpatient clinic, the patient began chemotherapy with gemcitabine and ascitic fluid gradually accumulated. Therefore, the patient was administered combination oral diuretic therapy with furosemide 20 mg/day and spironolactone 25 mg/day. Ascitic fluid continued to increase with chemotherapy and the patient's activities of daily living also declined, necessitating hospitalization for further examination.

The patient's height was 172 cm and his weight was 60 kg. His blood pressure 128/64 mmHg, and his pulse 96/min. The patient displayed abdominal distension, and intestinal peristaltic sound was heard. Blood test findings are shown in Table 1.

**Figure 2:**
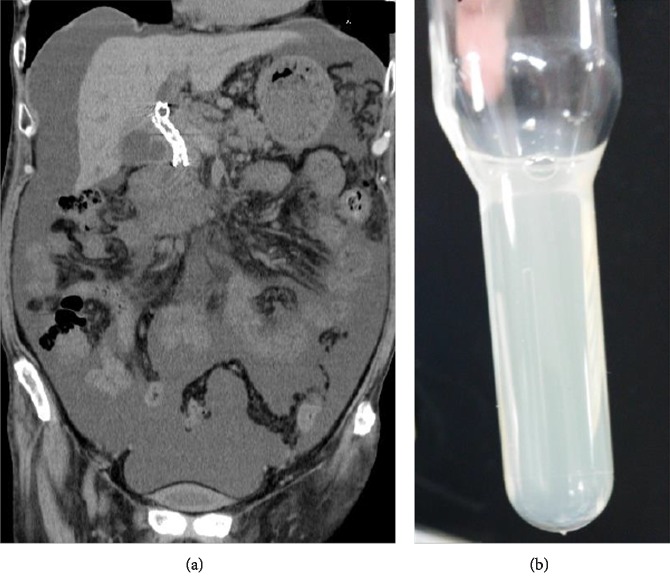
A large amount of ascites was stored in the abdominal CT (a). Harvested ascites (b).

An abdominal computed tomography (CT) image showed a large accumulation of ascitic fluid, and a test puncture revealed its milky appearance, and was indicative of chylous ascites (Figure 2).

The ascitic fluid was culture-negative, and cytology showed Class V of the Papanicolaou classification. In a biochemical examination of the ascites, TP (total protein) was lower but TGs were higher (Table 1). Because it was slightly lower than the definition provided by Wallis [9], we judged it as chylous ascites with a relatively low TG concentration. Initially, we reduced the NaCl and lipid content of the patient's diet to 5 g and 60 g per day, respectively, and continued treatment with furosemide and spironolactone, but we observed almost no decrease in ascetic fluid. Therefore, we stopped using medium-chain TGs (MCT) and reformulated the patient's diet to make it fat-restricted (1400 kcal, lipid 40 g per day) and added a single portion per day of Elental (300 kcal/80 g).

Elental was given orally and no side effects such as vomiting or diarrhea occurred.

**Figure 3:**
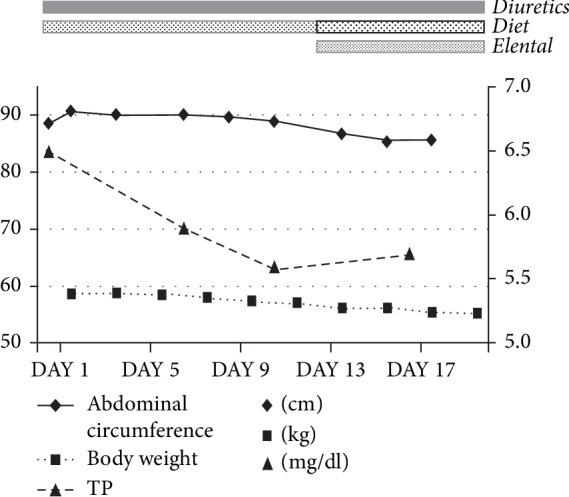
The body weight and abdominal girth decreased with the fat-restricted diet and the diuretic, Elental. At the same time, serum TP trended upward. TP, total protein.

Seven days after the change in diet, the patient's body weight decreased from 58 to 54 kg and his abdominal circumference decreased from 88 to 84 cm (Figure 3).

Chemotherapy was subsequently resumed at the outpatient clinic with tegafur/gimeracil/oteracil (TS-1). The ascitic fluid did not resolve completely but was attenuated by comparison with baseline status when managed via the oral administration of an elemental diet for approximately 1 month.

### 2.2. Case 2

An 82-year-old man with a history of cerebral infarction sequelae and dementia of the Alzheimer's type was treated for 2 weeks with antibiotics after a diagnosis of mesenteric panniculitis. One month after discharge from the hospital, the patient gained weight and experienced fatigue, upon which he visited the hospital. He was diagnosed with ascites, pleural effusion, and pericardial effusion, and he was then readmitted to the hospital.

The patient's height was 163 cm and his weight was 59.5 kg. There was no noise in the heart sound. Abdominal distension and lower leg edema were observed. Blood test findings were somewhat low (Table 2).

**Figure 4:**
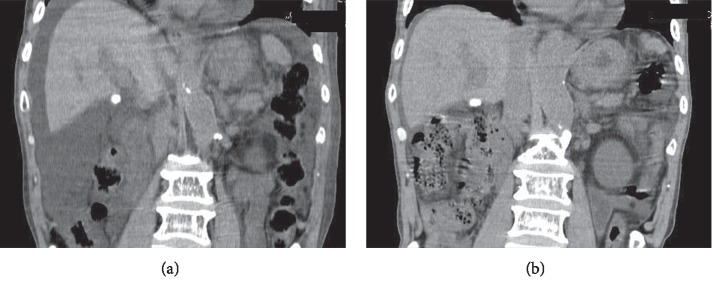
Abdominal CT showed massive storage of ascites (a) and a decrease in ascites after treatment (b).

An abdominal CT image showed a large amount of ascites (Figure 4(a)), and a test puncture revealed its milky appearance. A bacterial culture of the ascites was negative, and cytology showed it to be Class I of the Papanicolaou classification.

Initially, we placed the patient on a fat-restricted diet (1400 kcal and lipid 40 g per day), furosemide 10 mg/day, and a single dose of elemental diet, Elental, but improvements in weight and waist circumference were poor. Oral administration of Elental did not cause any side effects.

**Figure 5:**
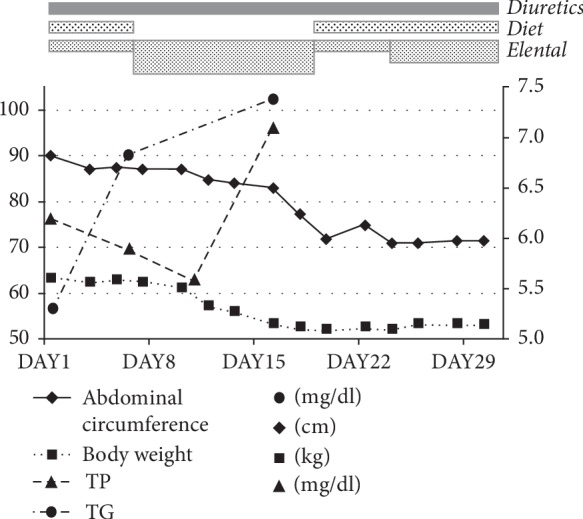
The body weight and abdominal girth decreased with the fat-restricted diet, the diuretic, and elental. At the same time, serum TG and TP increased.

After that, we stopped the fat-restricted diet and increased the amount of Elental to 3 times a day. In addition, peripheral parenteral nutrition was administered by infusion. The patient's body weight then decreased from 62 to 53 kg in 25 days, and his abdominal girth improved from 90 to 70 cm (Figure 5).

After that, we restarted the patient on a fat-restricted diet at 900 kcal divided into morning and evening, and lipid at 20 g/day, and the patient also consumed 2 bottles of Elental daily at noon.

Ultimately, oral nutrition and a small amount of diuretic administered orally resulted in better control of weight and abdominal girth. Abdominal CT also showed a marked improvement in ascites (Figure 4(b)). He was discharged from the hospital and continued treatment on an outpatient basis.

## 3. Discussion

Chylous ascites has a milky appearance and contains an abundant amount of TG, which leaches from the intestinal tract and the mesentery [1]. TGs ingested from meals are absorbed from the small intestine and flow into the portal vein via the lymph duct. However, if the lymphatic vessels in the abdominal cavity rupture and if TGs and lymph leak into the abdominal cavity from the collapsed site, chylous ascites occurs [1, 2, 10, 11]. If left untreated, the patient's general condition may deteriorate due to malnutrition and reduction of immunity. Therefore, appropriate treatment is necessary.

The causes of ascites are diverse, but the frequency of encountering chylous ascites is small. According to the report of Press et al., chylous ascites is found at a frequency of one case in 20,000 hospital cases, and of one case in 11,589 cases after intraperitoneal surgery [3]. It is a very rare condition. Most cases of chylous ascites occur after surgery [1], and the proportion of nonsurgical cases is low. It is said that malignancies (25%), liver cirrhosis (16%), and tuberculosis (15%) are among the most frequent causes of nonsurgical chylous ascites [4, 5]. Case 1 had bile duct cancer, and it was presumed that chylous ascites resulted from periportal lesion or peritoneal splenic lesion infiltrating the lymph duct [11].

In Case 2, mesenteric panniculitis spreading to lymph vessels was presumed to have caused the chylous ascites. In both cases, it was difficult to locate the site of failure in the lymphatic vessels using CT scans.

Chylous ascites may be treated by ligation of broken lymphatic vessels after surgery. However, by circumventing the intestinal tract, it is possible to prevent TGs from leaking into the abdominal cavity from the broken lymphatic vessels. First, it is recommended that primary management of chylous ascites should involve the placement of a central venous catheter in a fasting patient to provide TPN while bypassing the intestinal tract [2, 4, 6].

Secondary considerations may include MCTs and octreotide which have been mentioned as supplementary treatments. Since MCTs do not pass through the lymphatic system, but rather are absorbed directly into the portal vein according to several reports, MCTs do not increase lymph flow [2, 12, 13]. However, the response rate is less than 50%. Octreotide, a somatostatin analog, inhibits gut hormone secretion and exocrine secretions such as gastric juice, pancreatic juice, and bile. Octreotide treatment leads to control of nutrient absorption from the digestive tract, gastrointestinal motility, and secretion of lymph, which in turn promotes repair of the lymphatic system [5, 12]. However, the treatment of chylous ascites with octreotide is not covered by the current medical insurance plan in Japan.

Both patients in this report were elderly. The first patient had cancer with a poor prognosis, and the second patient had dementia such that there was a high risk of his pulling out the central venous catheter. In both cases, there were strong reasons to continue oral intake. To preserve quality of life, we treated the patients with an oral diet (fat-restricted diet and an elemental diet) before resorting to TPN via a central venous catheter.

There have been reports that parenteral nutrition and an oral fat-free elemental diet have been used as initial treatments for chylous ascites [7, 8]. In the patients in our report, although complete disappearance of ascites was not reached according to the imaging, weight, and abdominal girth decreased and quality of life improved.

In Case 1, blood TP improved from 5.6 to 5.7 mg/dl in 7 days. In Case 2, we were able to track improvements such as blood TG at 57–102 mg/dl in 18 days and TP at 5.6–7.1 mg/dl in 7 days.

Typical chylous ascites is defined as TG > 200 mg/dl [1, 3]. However, there are reports that set the cutoff value at ≥110 mg/dl [3], and those that report TG concentration in ascites as 2–8 times the serum TG concentration [5, 14]. Serum TG concentration was relatively low in the 2 cases at our hospital. We presumed that the ascites TG concentrations were low due to the fact that these cases were atraumatic and that the serum TG concentrations were low due to aging and malnutrition. According to the classification in the report by Wallis [9], cases with relatively low concentration of ascites TG are defined as pseudo-chylous ascites. In addition, according to the report of Cardenas and Chopra [15], such cases are considered as atypical chylous ascites. However, the treatment method for cases with a relatively low ascites TG concentration has not been discussed until our report.

Elental is an enteral high calorie nutritional supplement with amino acids. Elental was created in the process of developing easy-to-absorb food for astronauts and was then applied for medical use. It is administered to patients with poor oral intake, patients with hypoproteinemia and patients requiring nutritional management after surgery or for Crohn's disease to give the intestines respite for healing [16]. A preparation with a lower lipid concentration is also frequently used for conditions such as chronic or acute pancreatitis [17, 18]. Nakayama and colleagues reported on a patient who developed chylous ascites 3 weeks after surgery for advanced rectal cancer; the ascites disappeared 7 weeks after initiation of Elental at 30 kcal/kg/day [8].

The two patients in this report had non-postoperative, atraumatic chylous ascites. Among cases of chylous ascites, such cases are relatively rare. Atraumatic chylous ascites like these two cases may be difficult to treat by ligation of broken lymphatic vessels. The site of rupture of lymphatic vessels is unclear. The original disease makes it difficult to cure the broken site. Because of these two reasons, chylous ascites of our patients may have been managed only partially.

Elental was chosen because the patients had two needs: continuation of oral nutrition intake and fat restriction. Both patients showed good control of both weight and abdominal circumference by approximately 1–3 weeks after starting Elental. In the meantime, we observed serum TG and TP improvement.

It is unknown whether a specific type of chylous ascites can be managed with an oral elemental diet and a specific TG concentration. Furthermore, the amount and the period of use have not yet been defined. In order to clarify these points, future cases must be studied.

## 4. Conclusion

Fat restriction with Elental was provided with concurrent oral nutrition. Elental could be useful as a treatment option for chylous ascites.

## Figures and Tables

**Table 1:** Laboratory findings on admission (Case 1).

WBC	2700	/*μ*l	Glu	96	mg/dl
RBC	342	×10/^4^ *μ*l	Na	142	mEq/l
Hb	10.1	g/dl	K	4.2	mEq/l
Plt	16.7	×10/^4^ *μ*l	Cl	107	mEq/l
T-Bil	0.6	mg/dl	T-cho	144	mg/dl
AST	29	IU/l	TG	47	mg/dl
ALT	20	IU/l			
LDH	177	IU/l		Ascites	
ALP	399	IU/l	TP	0.8	g/dl
*γ*-GTP	41	IU/l	LDH	37.0	IU/l
AMY	50	IU/l	Na	139.7	mEq/l
TP	6.5	g/dl	K	3.8	mEq/l
Alb	2.9	g/dl	Cl	111.3	mEq/l
BUN	9.9	mg/dl	Glu	133	mg/dl
Cre	0.54	mg/dl	TG	48	mg/dl
CEA	92.1	ng/ml	Bacterial culture; negative		
CA19-9	<2.0	U/ml	Cytology; Class V		

Abbreviations: WBC: whole blood cell count, RBC: red blood cell count, Hb: hemoglobin, Plt: platelet count, T-Bil: total-value bilirubin, AST: aspartate transferase, ALT: alanine transaminase, LDH: lactate dehydrogenase, ALP: alkaline phosphatase, *γ*-GTP: gamma-glutamyl transferase, AMY: amylase, TP: total protein, Alb: albumin, BUN: blood urea nitrogen, CRE: creatinine, CEA: carcinoembryonic antigen, CA 19-9: cancer antigen 19-9, Glu: glucose, Na: sodium, K: potassium, Cl: chlorine, T-cho: total cholesterol, TG: triglycerides.

**Table 2:** Laboratory findings on admission (Case 2).

WBC	6000	/*μ*l	Na	136	mEq/l
RBC	346	×10/^4^ *μ*l	K	3.5	mEq/l
Hb	10.0	g/dl	Cl	107	mEq/l
Plt	24.6	×10/^4^ *μ*l	T-cho	166	mg/dl
T-Bil	0.27	mg/dl	TG	57	mg/dl
AST	15	IU/l			
ALT	9	IU/l		Ascites	
LDH	187	IU/l	TP	0.8	g/dl
ALP	358	IU/l	LDH	28.0	IU/l
*γ*-GTP	50	IU/l	Na	139.6	mEq/l
AMY	64	IU/l	K	3.4	mEq/l
TP	6.2	g/dl	Cl	116.5	mEq/l
BUN	13.9	mg/dl	Glu	193	mg/dl
Cre	1.53	mg/dl	TG	71	mg/dl
CEA	<0.5	ng/ml	Bacterial culture; negative		
Glu	113	mg/dl	Cytology; Class I		

Abbreviations: WBC: whole blood cell count, RBC: red blood cell count, Hb: hemoglobin, Plt: platelet count, T-Bil: total-value bilirubin, AST: aspartate transferase, ALT: alanine transaminase, LDH: lactate dehydrogenase, ALP: alkaline phosphatase, *γ*-GTP: gamma-glutamyl transferase, AMY: amylase, TP: total protein, BUN: blood urea nitrogen, CRE: creatinine, CEA: carcinoembryonic antigen, Glu: glucose, Na: sodium, K: potassium, Cl: chlorine, T-cho: total cholesterol, TG: triglycerides.
